# Golimumab in patients with active rheumatoid arthritis after treatment with tumor necrosis factor α inhibitors: findings with up to five years of treatment in the multicenter, randomized, double-blind, placebo-controlled, phase 3 GO-AFTER study

**DOI:** 10.1186/s13075-015-0516-6

**Published:** 2015-01-22

**Authors:** Josef S Smolen, Jonathan Kay, Mittie Doyle, Robert Landewé, Eric L Matteson, Norman Gaylis, Jürgen Wollenhaupt, Frederick T Murphy, Stephen Xu, Yiying Zhou, Elizabeth C Hsia

**Affiliations:** Department of Medicine, Medical University of Vienna, Hietzing Hospital, Vienna, Austria; Division of Rheumatology, Department of Medicine 3, Medical University of Vienna, Waehringer Guertel 18-20, Vienna, 1090 Austria; Department of Medicine III, Hietzing Hospital, Waehringer Guertel 18-20, Vienna, A-1090 Austria; University of Massachusetts Medical School and UMass Memorial Center, Rheumatology Center, Memorial Campus, 119 Belmont Street, Worcester, MA 01605 USA; Translational Medicine Group, Alexion Pharmaceuticals, 75 Sidney Street, Cambridge, MA 02139 USA; Academic Medical Center/University of Amsterdam & Atrium Medical Center Heerlen, Henri Dunantstraat 5, Heerlen, PC 6419 The Netherlands; Divisions of Rheumatology and Epidemiology, Mayo Clinic College of Medicine, 200 1st St. S.W., Rochester, MN 55905 USA; Arthritis & Rheumatic Disease Specialties, 21097 NE 27th Court, Suite 200, Aventura, FL 33180 USA; Schoen Klinik Hamburg Eilbek, Dehnhaide 120, Hamburg, 22081 Germany; Altoona Center of Clinical Research, 175 Meadowbrook Lane, PO Box 1018, Duncansville, PA 16635 USA; Division of Rheumatology, University of Pennsylvania Medical School and Hospital of the University of Pennsylvania, Perelman Center 1 South, 3400 Civic Center Blvd., Philadelphia, PA 19104 USA; Janssen Research & Development, LLC, 1400 McKean Road, PO Box 776, Spring House, PA 19477 USA

## Abstract

**Introduction:**

The aim of this study was to assess long-term golimumab therapy in rheumatoid arthritis (RA) patients who discontinued previous tumor necrosis factor-α (TNF)-inhibitor(s).

**Methods:**

Patients enrolled into this multicenter, randomized, double-blind, placebo-controlled study of active RA (≥4 tender, ≥4 swollen joints) received placebo (Group 1) or golimumab 50 mg (Group 2) or 100 mg (Group 3) injections every 4 weeks. Patients in Groups 1 and 2 with inadequate response at week 16 escaped to golimumab 50 and 100 mg, respectively. At week 24, Group 1 patients crossed-over to golimumab 50 mg, Group 2 continued golimumab 50/100 mg per escape status, and Group 3 maintained dosing. During the long-term-extension (LTE), golimumab 50 mg could be increased to 100 mg, and 100 mg could be decreased to 50 mg. Data through 5 years are reported for all patients (safety) and patients using methotrexate (efficacy, intention-to-treat (ITT) analysis with last-observation-carried-forward for missing data and non-responder imputation for unsatisfactory efficacy discontinuations).

**Results:**

In total, 459 of 461 randomized patients received the study agent, 304 of whom were methotrexate-treated and included in efficacy analyses. Through week 256, the proportions of methotrexate-treated patients achieving American-College-of-Rheumatology (ACR) responses were 37.6% to 47.0% for ACR20, 21.4% to 35.0% for ACR50, and 7.8% to 17.0% for ACR70 response across randomized groups. Golimumab safety through week 268 was generally consistent with that at week 24 and week 160 and other anti-TNF agents.

**Conclusions:**

In some patients with active RA discontinuing previous TNF-antagonist therapy, golimumab safety and efficacy, assessed conservatively with ITT analyses, was confirmed through 5 years.

**Trial registration:**

Clinicaltrials.gov NCT00299546. Registered 03 March 2006.

**Electronic supplementary material:**

The online version of this article (doi:10.1186/s13075-015-0516-6) contains supplementary material, which is available to authorized users.

## Introduction

The GOlimumab After Former anti-tumor necrosis factor α Therapy Evaluated in Rheumatoid arthritis (GO-AFTER) study (Clinicaltrials.gov NCT00299546; registered 3 March 2006) was the first and hitherto only prospective, randomized, phase 3, double-blind, placebo-controlled trial to assess a tumor necrosis factor (TNF) α inhibitor exclusively in patients with active rheumatoid arthritis (RA) who previously received TNF inhibitor(s). Patients had also received several disease-modifying antirheumatic drugs prior to TNF inhibitor(s), thereby representing a difficult-to-treat population. As reported previously, treatment with golimumab 50 mg or 100 mg every 4 weeks yielded significantly higher response rates for ≥20% improvement in the American College of Rheumatology criteria (ACR20) than treatment with placebo at week 14 [1,2]. At week 160 of the GO-AFTER trial, golimumab 50 mg and 100 mg injections every 4 weeks resulted in persistent improvement in signs and symptoms of RA and physical function among patients who continued therapy throughout this observation period of 3 years [2].

Long-term extension (LTE) phases of clinical trials typically are associated with special concerns in data reporting because of the bias resulting from assessment only of patients who were responding to treatment and who continued study participation [3]. However, both patients and providers can benefit from assessing the outcome of patients who respond to treatment as well as the outcome for all patients who started a specific therapy. Needless to say, it is particularly challenging for patients with disease refractory to several prior therapies – including biological agents, as was the case for the GO-AFTER study population [1,2] – to achieve and maintain clinical responses.

The GO-AFTER study was designed to include a LTE phase of golimumab therapy. The 5-year data, which comprise the entire planned trial, are reported herein and include information about long-term safety in this patient population.

## Methods

The GO-AFTER study was conducted according to the Declaration of Helsinki. All patients provided written informed consent, and the protocol was approved by each institution’s ethical review board (see Acknowledgements for details).

Details of the GO-AFTER patients with RA [4] and the study methods have been reported previously; procedures and analyses specific to the LTE, including assessments of clinical response, quality of life, safety and immunogenicity [5-14], are summarized in Additional file 1.

## Results

### Patient disposition and baseline patient and disease characteristics

Patient disposition through week 24 [1] and week 160 [2] of the GO-AFTER trial has been reported previously. Through week 252, 276 (60.1%) patients discontinued the study agent (Figure S1 in Additional file 1), most commonly because of unsatisfactory therapeutic effect (*n* = 107), adverse events (*n* = 86), and other reasons (*n* = 69). The proportions of patients discontinuing the study agent due to adverse events or unsatisfactory therapeutic effect increased with a greater number of TNF antagonists taken (data not shown). Baseline methotrexate (MTX) use was reported by 311 treated patients. Among these, 58.2% (181/311) discontinued the study agent. In patients receiving golimumab monotherapy, 64.2% (95/148) discontinued the study agent. Baseline patient/disease characteristics have been reported [1,2] and are summarized in Table S1 in Additional file 1. During the LTE, 139 patients escalated the golimumab dose (from 50 mg to 100 mg) and 29 patients reduced the dose (from 100 mg to 50 mg) at the investigator’s discretion (Figure S1 in Additional file 1).

### Clinical outcomes

As reported previously, at week 24 the proportions of all patients achieving ACR20 response, ≥50% improvement in the American College of Rheumatology criteria (ACR50) response, 28-joint Disease Activity Score (DAS28) response and DAS28 score <2.6 among patients who received golimumab 50 mg and 100 mg were significantly higher than for placebo-treated patients (all *P* <0.05) [1].

**Figure 1:**
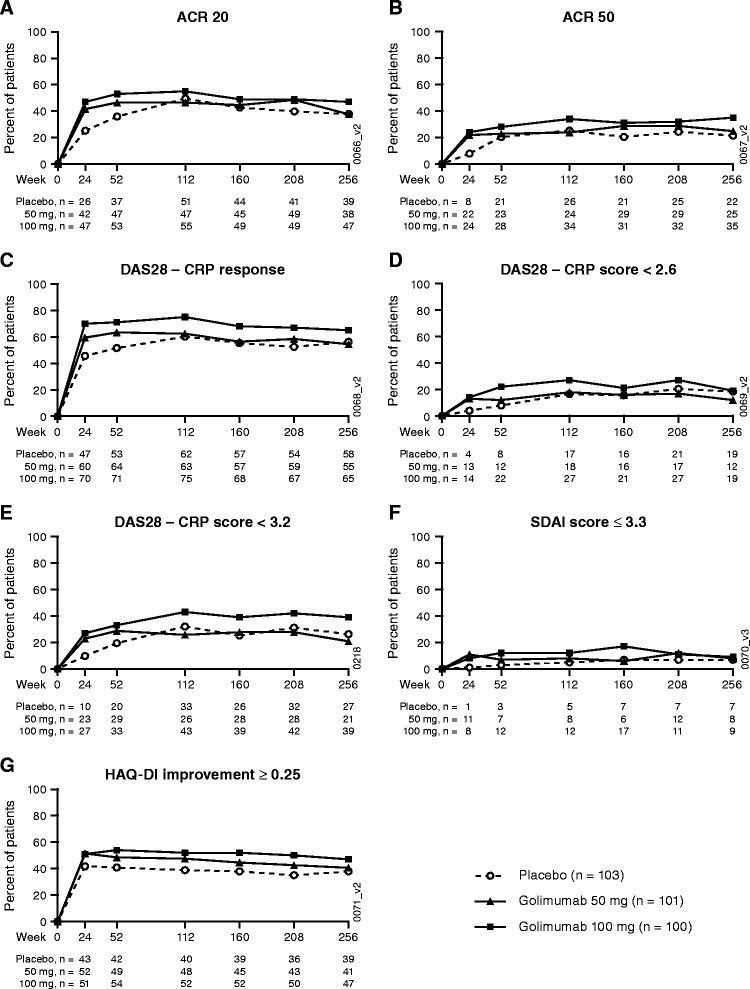
**Clinical efficacy over time through week 256.** ACR20 **(A)**, ACR50 **(B)**, DAS28-CRP response **(C)**, DAS28-CRP score <2.6 **(D)**, DAS28-CRP score <3.2 **(E)**, SDAI score ≤3.3 **(F)**, and HAQ-DI improvement ≥0.25 **(G)**. Data summarized for randomized patients receiving methotrexate at baseline, excluding one site, using intent-to-treat methodology, with replacement of missing data by last-observation-carried forward methodology and imputation with baseline median values, and nonresponder imputation for discontinuations due to unsatisfactory therapeutic effect. ACR20/50, ≥20%/50% improvement in the American College of Rheumatology response criteria; CRP, C-reactive protein; DAS28, 28-joint Disease Activity Score; HAQ-DI, Health Assessment Questionnaire Disability Index; SDAI, Simplified Disease Activity Index*.*

Clinical outcomes through 5 years are primarily summarized using an intent-to-treat analysis. Given that all patients received golimumab from week 16 or 24, no treatment group comparisons were undertaken. Based on intent-to-treat efficacy data, the proportions of MTX-treated patients who achieved ACR20, ACR50, DAS28 employing C-reactive protein (DAS28-CRP) response, and DAS28-CRP scores <2.6 and <3.2 were consistent through week 256. At this time, 37.9% (39/103) of patients were randomized to receive placebo and then golimumab from week 16 (early escape) or week 24 onwards: 42.3% (85/201) of golimumab-randomized patients achieved ACR20 response, 21.4% (22/103) and 29.9% (60/201), respectively, achieved ACR50 response, 56.3% (58/103) and 59.7% (120/201) achieved DAS28-CRP response, 18.4% (19/103) and 15.4% (31/201) achieved DAS28-CRP <2.6, and 26.2% (27/103) and 29.9% (60/201) achieved DAS28-CRP <3.2 (Figure 1A,B,C,D,E). Clinical remission, defined as Simplified Disease Activity Index ≤3.3, was achieved by 6.8% (7/103) and 8.5% (17/201) of placebo-randomized and golimumab-randomized patients, respectively, at week 256 (Figure 1F). At week 256, 37.9% (39/103) and 43.8% (88/201) of patients, respectively, achieved ≥0.25 unit improvement in the Health Assessment Questionnaire Disability Index score (Figure 1G). Similar trends were evident when responses were assessed as observed data, albeit at higher rates due to the completer nature of those analyses (Figure S2 in Additional file 1).

### Immunogenicity

The overall cumulative incidence of antibodies to golimumab through week 268 was low (8.0%) and increased only slightly over time. Most of these patients tested positive for neutralizing antibodies (Table 1).

**Table 1:** **Cumulative summary of golimumab safety and immunogenicity through week 268 of the GO-AFTER trial**

	**Golimumab**			
	**50 mg only**	**50 and 100 mg**	**100 mg only**	**All patients**
Number of treated patients	98	195	138	431
Mean duration of follow-up (weeks)	129.82	187.45	162.06	166.22
Mean number of injections	29.4	42.9	37.0	37.9
Patients with one or more adverse events	90 (91.8%)	186 (95.4%)	132 (95.7%)	408 (94.7%)
Common adverse events^a^				
Upper respiratory tract infection	25 (25.5%)	49 (25.1%)	43 (31.2%)	117 (27.1%)
Rheumatoid arthritis	17 (17.3%)	57 (29.2%)	25 (18.1%)	99 (23.0%)
Nasopharyngitis	10 (10.2%)	37 (19.0%)	26 (18.8%)	73 (16.9%)
Sinusitis	19 (19.4%)	35 (17.9%)	23 (16.7%)	77 (17.9%)
Back pain	8 (8.2%)	36 (18.5%)	18 (13.0%)	62 (14.4%)
Hypertension	10 (10.2%)	34 (17.4%)	17 (12.3%)	61 (14.2%)
Arthrlagia	13 (13.3%)	26 (13.3%)	21 (15.2%)	60 (13.9%)
Bronchitis	12 (12.2%)	24 (12.3%)	22 (15.9%)	58 (13.5%)
Diarrhea	5 (5.1%)	28 (14.4%)	22 (15.9%)	55 (12.8%)
Urinary tract infection	13 (13.3%)	25 (12.8%)	13 (9.4%)	51 (11.8%)
Nausea	10 (10.2%)	21 (10.8%)	18 (13.0%)	49 (11.4%)
Headache	14 (14.3%)	19 (9.7%)	14 (10.1%)	47 (10.9%)
Cough	10 (10.2%)	24 (12.3%)	13 (9.4%)	47 (10.9%)
Death				
Observed number of patients	2 (2.0%)	6 (3.1%)	1 (0.7%)	9 (2.1%)
Incidence (95% CI)/100 pt-yrs^b^	0.82 (0.10, 2.95)	0.85 (0.31, 1.86)	0.23 (0.01, 1.30)	0.65 (0.30, 1.24)
Discontinuation due to adverse event(s)	24 (24.5%)	33 (16.9%)	24 (17.4%)	81 (18.8%)
Serious adverse events	34 (34.7%)	71 (36.4%)	46 (33.3%)	151 (35.0%)
Common serious adverse events^c^				
Pneumonia	3 (3.1%)	10 (5.1%)	5 (3.6%)	18 (4.2%)
Urinary tract infection	0	5 (2.6%)	2 (1.4%)	7 (1.6%)
Rheumatoid arthritis	4 (4.1%)	8 (4.1%)	2 (1.4%)	14 (3.2%)
Osteoarthritis	2 (2.0%)	8 (4.1%)	1 (0.7%)	11 (2.6%)
Sepsis	0	5 (2.6%)	1 (0.7%)	6 (1.4%)
Arthralgia	1 (1.0%)	1 (0.5%)	2 (1.4%)	4 (0.9%)
Infections	64 (65.3%)	149 (76.4%)	108 (78.3%)	321 (74.5%)
Serious infections				
Observed number of patients	12 (12.2%)	29 (14.9%)	19 (13.8%)	60 (13.9%)
Observed number of serious infections	16	46	35	97
Incidence (95% CI)/100 pt-yrs^d^	6.54 (3.74, 10.62)	6.54 (4.79, 8.73)	8.14 (5.67, 11.32)	7.04 (5.71, 8.59)
Common serious infections^e^				
Pneumonia	3 (3.1%)	10 (5.1%)	5 (3.6%)	18 (4.2%)
Urinary tract infection	0	5 (2.6%)	2 (1.4%)	7 (1.6%)
Sepsis	0	5 (2.6%)	1 (0.7%)	6 (1.4%)
Cellulitis	1 (1.0%)	2 (1.0%)	1 (0.7%)	4 (0.9%)
Diverticulitis	0	1 (0.5%)	2 (1.4%)	3 (0.7%)
Pneumonitis	1 (1.0%)	0	1 (0.7%)	2 (0.5%)
Colitis ulcerative	1 (1.0%)	0	0	1 (0.2%)
Diarrhea	1 (1.0%)	0	0	1 (0.2%)
Vomiting	1 (1.0%)	0	0	1 (0.2%)
Golimumab injection-site reactions				
Patients with reactions	11 (11.2%)	24 (12.3%)	18 (13.0%)	53 (12.3%)
Injections with reactions	16 (0.6%)	49 (0.6%)	64 (1.3%)	129 (0.8%)
Antibodies to golimumab				
Week 52	5 (5.4%)	9 (5.4%)	6 (4.6%)	20 (5.2%)
% with neutralizing antibodies^f^	3/4 (75.0%)	5/5 (100.0%)	6/6 (100.0%)	14/15 (93.3%)
Week 100	6 (6.5%)	12 (7.2%)	7 (5.4%)	25 (6.4%)
% with neutralizing antibodies^f^	4/5 (80.0%)	10/10 (100.0%)	6/7 (85.7%)	20/22 (90.9%)
Week 268	7 (7.6%)	16 (9.6%)	8 (6.2%)	31 (8.0%)
% with neutralizing antibodies^f^	5/6 (83.3%)	14/14 (100.0%)	6/8 (75.0%)	25/28 (89.3%)

Data presented are number (%) of patients unless noted otherwise. CI, confidence interval (based on exact method); GO-AFTER, GOlimumab After Former anti-tumor necrosis factor α Therapy Evaluated in Rheumatoid arthritis; pt-yrs, patient-years of follow-up. ^a^Occurring in ≥10% of patients in the combined golimumab group. ^b^The incidence of death for placebo through week 24 was 0.00 (95% CI: 0.00, 6.20). ^c^Occurring in ≥1% of patients in any golimumab group. ^d^The incidence of serious infections for placebo through week 24 was 2.07 (95% CI: 0.05, 11.52). ^e^Occurring in ≥1% of patients in any golimumab group. ^f^Among patients with samples evaluable for testing.

### Adverse events

Adverse events through week 24 and week 160 of the GO-AFTER trial have been reported previously [1,2]. Eleven patients died through week 268, including one placebo-treated patient who died of pancreatic cancer during the 24-week study period [1] and 10 golimumab-treated patients who died after week 24 (Table 1). No predominant cause of death was identified throughout the 5-year trial (see Additional file 1).

Serious adverse events were reported for approximately one-third of golimumab-treated patients, with the most common categorized as infections. Infections were also the most common adverse events leading to study agent discontinuation (Table 1). The overall pattern and types of infections observed through week 268 were similar to those reported through week 24 [1]. Through week 268, 13.9% of patients in the combined golimumab group had ≥1 infection identified by the investigator as a serious adverse event (Table 1). One case of active tuberculosis (pulmonary) was reported for a patient who was receiving golimumab 100 mg. Histoplasmosis infection occurred in two patients, each judged to be a serious infectious event (one disseminated, with both patients receiving golimumab 100 mg at event onset). Four patients had opportunistic infections through week 268, including three patients with esophageal candidiasis (one patient who was receiving 50 mg, two patients who were receiving 100 mg) and one with ophthalmic herpes zoster (100 mg).

Twenty patients in the combined golimumab-treated group had malignancies reported through week 268, including lymphoma (four patients who were receiving golimumab 100 mg) and nonmelanoma skin cancers. The 95% confidence intervals (CIs) surrounding the incidence/100 patient-years of follow-up of all malignancy categories observed with golimumab were contained within the 95% CI for placebo through week 24 (that is, 0.00 (0.00, 6.20)). The Standardized Incidence Ratio and surrounding 95% CI for lymphoma indicated a higher than expected occurrence among patients who received golimumab 100 mg; however, the Standardized Incidence Ratios (95% CI) for all other malignancies (excluding nonmelanoma skin cancers, which are not captured in the Surveillance, Epidemiology, and End Results database) indicated no increased risk relative to the general US population (Table 2). See Additional file 1 for additional safety findings.

**Table 2:** **Number of patients with one or more malignancies through week 268 compared with the expected number of malignancies from the general US population according to the SEER database**

	**Golimumab**			
	**50 mg only**	**50 and 100 mg**	**100 mg only**	**All patients**
Treated patients in the study	98	195	138	431
**All malignancies**				
Observed number of patients	3	11	6	20
Incidence (95% CI)/100 pt-yrs^a^	1.23 (0.25, 3.59)	1.59 (0.79, 2.85)	1.42 (0.52, 3.10)	1.47 (0.90, 2.28)
SIR^b^	1.36	1.20	0.81	1.11
(95% CI)^c^	(0.28, 3.98)	(0.48, 2.47)	(0.17, 2.36)	(0.59, 1.89)
**Type of malignancy**				
Lymphoma				
Observed number of patients	0	2	2	4
Incidence (95% CI)/100 pt-yrs^a^	0.00 (0.00, 1.22)	0.28 (0.03, 1.03)	0.47 (0.06, 1.68)	0.29 (0.08, 0.74)
SIR^b^	0.00	8.13	12.32	7.96
(95% CI)^c^	(0.00, 31.87)	(0.99, 29.38)	(1.49, 44.49)	(2.17, 20.39)
Other malignancies				
Observed number of patients	3	5	1	9
Incidence (95% CI)/100 pt-yrs^a^	1.23 (0.25, 3.59)	0.71 (0.23, 1.66)	0.23 (0.01, 1.30)	0.65 (0.30, 1.24)
SIR^b^	1.42	0.89	0.28	0.80
(95% CI)^c^	(0.29, 4.15)	(0.29, 2.07)	(0.01, 1.56)	(0.36, 1.51)
Nonmelanoma skin cancer				
Observed number of patients	0	5	3	8
Incidence (95% CI)/100 pt-yrs^a^	0.00 (0.00, 1.22)	0.72 (0.23, 1.69)	0.71 (0.15, 2.08)	0.59 (0.25, 1.16)

CI, confidence interval; pt-yrs, patient-years of follow-up; SEER, Surveillance, Epidemiology, and End Results. ^a^The incidences (95% CIs) for placebo through week 24 were 0.00 (0.00, 6.20) for lymphoma, 0.00 (0.00, 6.20) for nonmelanoma skin cancer, 0.00 (0.00, 6.20) for other malignancies, and 0.00 (0.00, 6.20) for all malignancies. ^b^Standardized Incidence Ratio (SIR) (observed number of patients with malignancy based on the SEER database [14], adjusted for age, gender, and race divided by expected number of patients with malignancy). SIRs (95% CIs) for placebo through week 24 were 0.00 (0.00, 175.37) for lymphoma, 0.00 (0.00, 7.82) for other malignancies, and 0.00 (0.00, 7.51) for all malignancies. ^b^Confidence intervals based on an exact method.

## Discussion

The GO-AFTER trial evaluated patients with active RA despite prior treatment with conventional synthetic disease-modifying antirheumatic drugs and ≥1 TNF inhibitor(s) (a particularly treatment-refractory cohort with longstanding disease) for their response to yet another TNF inhibitor, golimumab. The LTE data presented reveal that, despite refractory disease, 40% of randomized patients continued in the study through 5 years. Among completers, >50% of patients randomized to golimumab plus MTX maintained low disease activity according to DAS28 criteria and 15% attained remission according to stringent American College of Rheumatology–European League Against Rheumatism index-based criteria. Among all randomized patients, >20% achieved low disease activity and approximately 8% achieved stringent remission criteria. For this treatment-resistant population, among whom approximately one-third had not received MTX and thus was less amenable to responding versus combination therapy [15], this was not necessarily expected. The data show that the TNF inhibitor golimumab can indeed exert sustained significant efficacy in some patients who previously discontinued ≥1 TNF inhibitor.

Similar LTE data have not been published for other biological agents – for example, tocilizumab, rituximab, abatacept, and other TNF inhibitors. Indirect comparisons cannot therefore be made. However, we have shown previously that 6-month response rates to golimumab plus MTX were similar to those of other targeted biologics in similar patient populations [1].

There are several limitations to the analyses after week 24. No patients received placebo after week 24, yielding no control group after this time point. The study drug was administered open-label after the week 24 database lock. Patients could change golimumab treatment from 50 mg to 100 mg (*n* = 139) and from 100 mg to 50 mg (*n* = 29) during the LTE according to investigator judgment. These uncontrolled dose changes limit conclusions regarding the effect of dose change. These changes in treatment regimens also hinder comparisons between the golimumab 50 mg and 100 mg dose groups. Exposure to golimumab 100 mg was substantially greater, both in number of patients and length of follow-up. Patients with more severe RA disease, who would probably be more difficult to treat and more prone to experience adverse events, may have been selectively escalated to the higher dose. These confounding factors preclude drawing definitive conclusions regarding the relative dose comparability.

The safety data revealed no new findings as compared with earlier phases of the GO-AFTER trial [1,2], with serious infections occurring in 14% of the patients. When adjusted for length of follow-up, the incidence of serious infections among all golimumab-treated patients was 7.04/100 patient-years of follow-up, which is consistent with those observed in a retrospective observational population-based inception cohort of patients diagnosed with RA between 1995 and 2007; that is, 6.6/100 patient-years of follow-up for all patients and 8.2/100 patient-years of follow-up during treatment with biologic agents [16]. Of note, four patients treated with 100 mg developed lymphoma, a rate significantly higher than expected; all occurred within the first 3 years of observation [2]. No patient in the 50 mg group developed lymphoma. Lymphoma is associated with cumulative RA disease activity [17] and registry data have not shown an association between TNF inhibitor treatment and increased lymphoma risk [18]. When adjusted for length of follow-up, the overall incidence of lymphoma reported herein (0.29/100 patient-years of follow-up) was higher than that previously reported based on a longitudinal (1998 to 2005) study of long-term outcomes of RA patients (0.11/100 patient-years of follow-up) [19]. However, due to the relatively small numbers of events and patients in our trial, the accompanying 95% CI was fairly wide (95% CI: 0.08, 0.74) and in fact overlapped that of the longitudinal trial which evaluated 19,591 patients for 89,710 patient-years of follow-up (95% CI: 0.09, 0.13) [19]. Whether the increased rate we observed for the 100 mg dose is related to the drug dose itself or to the theoretically higher cumulative disease activity of patients receiving this dose due to dose escalation (see above and as discussed elsewhere [2]) remains unknown.

## Conclusion

Golimumab can be an effective therapy over the long term for RA patients who have previously received and discontinued another TNF inhibitor therapy for reasons including insufficient efficacy. Almost 40% of the patients originally randomized to golimumab continued therapy for 5 years and many achieved low disease activity/remission with this treatment, despite their refractory disease.

## Additional file

Additional file 1:
**Is a word file providing further details of study methods, patient disposition and baseline characteristics, and additional efficacy and safety findings.**

## Abbreviations

ACR20/50: ≥20/50% improvement in the American College of Rheumatology criteria
CI: confidence interval
DAS28: 28-joint count Disease Activity Score
CRP: C-reactive protein
GO-AFTER: GOlimumab After Former anti-tumor necrosis factor α Therapy Evaluated in Rheumatoid arthritis
LTE: long-term extension
MTX: methotrexate
RA: rheumatoid arthritis
TNF: tumor necrosis factor

## References

[1] Smolen JS, Kay J, Doyle MK, Landewé R, Matteson EL, Wollenhaupt J (2009). GO-AFTER study investigators: golimumab in patients with active rheumatoid arthritis after treatment with tumour necrosis factor α inhibitors (GO-AFTER study): a multicentre, randomised, double-blind, placebo-controlled, phase III trial. Lancet.

[2] Smolen JS, Kay J, Landewé RB, Matteson EL, Gaylis N, Wollenhaupt J (2012). Golimumab in patients with active rheumatoid arthritis who have previous experience with tumour necrosis factor inhibitors: results of a long-term extension of the randomised, double-blind, placebo-controlled GO-AFTER study through week 160. Ann Rheum Dis.

[3] Buch MH, Aletaha D, Emery P, Smolen JS (2011). Reporting of long-term extension studies: lack of consistency calls for consensus. Ann Rheum Dis.

[4] Arnett FC, Edworthy SM, Bloch DA, McShane DJ, Fries JF, Cooper NS (1988). The American Rheumatism Association 1987 revised criteria for the classification of rheumatoid arthritis. Arthritis Rheum.

[5] Felson DT, Anderson JJ, Boers M, Bombardier C, Furst D, Goldsmith C (1995). American College of Rheumatology: preliminary definition of improvement in rheumatoid arthritis. Arthritis Rheum.

[6] Prevoo ML, van’t Hof MA, Kuper HH, van Leeuwen MA, van de Putte LB, van Riel PL (1995). Modified disease activity scores that include twenty-eight-joint-counts. Development and validation in a prospective longitudinal study of patients with rheumatoid arthritis. Arthritis Rheum.

[7] Aletaha D, Landewe R, Karonitsch T, Bathon J, Boers M, Bombardier C (2008). Reporting disease activity in clinical trials of patients with rheumatoid arthritis: EULAR/ACR collaborative recommendations. Arthritis Rheum.

[8] van Riel PL, van Gestel AM, Scott DL (2000). EULAR handbook of clinical assessments in rheumatoid arthritis.

[9] Wells G, Becker JC, Teng J, Dougados M, Schiff M, Smolen J (2009). Validation of the 28-joint Disease Activity Score (DAS28) and European League Against Rheumatism response criteria based on C-reactive protein against disease progression in patients with rheumatoid arthritis, and comparison with the DAS28 based on erythrocyte sedimentation rate. Ann Rheum Dis.

[10] Smolen JS, Breedveld FC, Schiff MH, Kalden JR, Emery P, Eberl G (2003). A simplified disease activity index for rheumatoid arthritis for use in clinical practice. Rheumatology.

[11] Felson DT, Smolen JS, Wells G, Zhang B, van Tuyl LH, Funovits J (2011). American College of Rheumatology/European League Against Rheumatism provisional definition of remission in rheumatoid arthritis for clinical trials. Arthritis Rheum.

[12] Fries JF, Spitz P, Kraines RG, Holman HR (1980). Measurement of patient outcome in arthritis. Arthritis Rheum.

[13] Wells GA, Tugwell P, Kraag GR, Baker PR, Groh J, Redelmeier DA (1993). Minimum important difference between patients with rheumatoid arthritis: the patient’s perspective. J Rheumatol.

[14] National Cancer Institute. The Surveillance, Epidemiology, and End Results (SEER). http://seer.cancer.gov (2004).

[15] Smolen JS, Landewé R, Breedveld FC, Dougados M, Emery P, Gaujoux-Viala C (2014). EULAR recommendations for the management of rheumatoid arthritis with synthetic and biological disease-modifying antirheumatic drugs: 2013 update. Ann Rheum Dis.

[16] Ni Mhuircheartaigh OM, Matteson EL, Green AB, Crowson CS (2013). Trends in serious infections in rheumatoid arthritis. J Rheumatol.

[17] Baecklund E, Iliadou A, Askling J, Ekbom A, Backlin C, Granath F (2006). Association of chronic inflammation, not its treatment, with increased lymphoma risk in rheumatoid arthritis. Arthritis Rheum.

[18] Askling J, van Vollenhoven RF, Granath F, Raaschou P, Fored CM, Baecklund E (2009). Cancer risk in patients with rheumatoid arthritis treated with anti-tumor necrosis factor α therapies: does the risk change with the time since start of treatment?. Arthritis Rheum.

[19] Wolfe F, Michaud K (2007). The effect of methotrexate and anti-tumor necrosis factor therapy on the risk of lymphoma in rheumatoid arthritis in 19,562 patients during 89,710 person-years of observation. Arthritis Rheum.

