# Supplementary material for: Golimumab in patients with active rheumatoid arthritis after treatment with tumor necrosis factor α inhibitors: findings with up to five years of treatment in the multicenter, randomized, double-blind, placebo-controlled, phase 3 GO-AFTER study
Source: Arthritis Res Ther. 2015 Jan 22;17(1):14. doi: 10.1186/s13075-015-0516-6 (PMC4382834; doi:10.1186/s13075-015-0516-6)
Supplement: Additional file 1: — Is a word file providing further details of study methods, patient disposition and baseline characteristics, and additional efficacy and safety findings. [file 13075_2015_516_MOESM1_ESM.docx]

**ADDITIONAL FILE 1**

**Patients and methods**

Patient enrollment began February 21, 2006; data were collected at visits conducted through May 21, 2012 as part of the trial’s long-term extension. Patients with rheumatoid arthritis[4] were eligible if they had active disease (≥ 4 swollen, ≥ 4 tender joints). Patients had previously received etanercept or adalimumab for at least 8 weeks or infliximab for at least 12 weeks and could have discontinued these agents for any reason (documented as lack of efficacy, intolerance, or other). Additional inclusion/exclusion criteria were previously reported.[1]

Patients were randomized (1:1:1) to receive subcutaneous placebo, golimumab 50 mg, or golimumab 100 mg every 4 weeks. Stable doses of synthetic disease-modifying antirheumatic drugs were allowed. Patients and investigators were blinded to treatment assignment; golimumab and placebo were supplied in identical single-use vials.

Patients in the placebo and golimumab 50-mg groups with < 20% improvement in both tender and swollen joint counts at week 16 early escaped to receive golimumab 50 mg or 100 mg, respectively, at week 16 and week 20. Study medication dose was not changed in the 100-mg group. GO-AFTER included a long-term extension from week 24 forward, during which patients in the placebo group crossed over to golimumab 50 mg every 4 weeks and patients in the golimumab 50-mg group continued with golimumab 50 mg or 100 mg every 4 weeks per their early escape status. The study blind was maintained during the long-term extension until the week 24 database lock, after which the golimumab dose could be either escalated from 50 mg to 100 mg or decreased from 100 mg to 50 mg at the investigator’s discretion. Following the last study golimumab injection, patients could transition to standard-of-care RA treatment at the discretion of their physicians.

Clinical response during the long-term extension was assessed using American College of Rheumatology (ACR)[5] and 28-joint count Disease Activity Score (DAS28) response criteria (good/moderate response and DAS28 scores < 2.6 and < 3.2).[6-9] Clinical remission according to ACR-European League Against Rheumatism (EULAR) criteria, evaluated using the Simplified Disease Activity Index (SDAI) score (≤3.3)[10,11], was determined post hoc. Physical function was assessed using the Health Assessment Questionnaire-Disability Index (HAQ-DI),[12] including achievement of a ≥ 0.25-unit improvement.[13] Adverse events were coded according to Medical Dictionary for Regulatory Activities (MedDRA).[1]

Clinical outcomes through 5 years are primarily summarized using an intent-to-treat analysis. Missing values for components of efficacy endpoints are imputed, using median group values as determined by the patient’s methotrexate stratification (yes/no) at baseline and last-observation-carried-forward methodology at all other time points. Patients who discontinued due to insufficient response were considered treatment failures, or nonresponders, from the time of treatment failure going forward. All patients remaining in the study, including those randomized to placebo, received golimumab with or without methotrexate for ≥4 years by study completion. Given that golimumab is approved for treatment of RA in combination with methotrexate, efficacy analyses include methotrexate-treated patients according to randomized treatment group. Observed efficacy data are also reported among methotrexate-treated patients who did not discontinue study participation as of week 24; no treatment failure rules were applied and missing data were not imputed in these analyses.

Efficacy data from one North American site that enrolled 16 patients were excluded because of protocol violations identified during standard audit processes. Patient baseline and safety data from these patients were not excluded.

Summaries of safety include data from randomized and treated patients. In this analysis, reported adverse events were summarized through week 268, with the exception of those occurring after receipt of any commercial biologic (including commercial golimumab), which were excluded from the safety summaries reported herein. Standardized incidence ratios for malignancies were determined using the Surveillance, Epidemiology and End Results database.[14]

**Safety results**

Deaths included one patient who died due to pancreatic cancer while receiving placebo during the 24-week study period[1] and 10 golimumab-treated patients who died after week 24. Among golimumab-treated patients, two died while receiving golimumab 50 mg (due to pneumonitis approximately 2 months after the last golimumab injection and stage 4 lung cancer approximately 2.5 months after the last golimumab injection) and seven died while receiving golimumab 100 mg (two patients due to pneumonia; one case described previously[2] and the other approximately 3 months following the last golimumab injection); and one patient each due to lymphoma/multi‑organ failure[2], diffuse large B-cell lymphoma approximately 1 month after the last golimumab injection, T-cell leukemia approximately 8 months after the last golimumab injection, cardiovascular event[2], and cardiomyopathy[2]). The last death was related to non-small cell lung carcinoma that occurred 4 months after the golimumab injection.

Demyelination occurred in two patients (both receiving 100 mg) through week 268, one of whom may have had signs/symptoms prior to study entry by retrospective medical history. Also through week 268, 12.3% of golimumab-treated patients had an injection-site reaction (Table 1). The most commonly reported injection-site reactions were erythema, pruritus, hematoma, and pain; no injection-site reaction was severe, serious, or resulted in study agent discontinuation. No events of anaphylaxis or serum sickness-like reactions were reported. Laboratory safety assessments through week 256 did not identify safety issues previously unreported through week 24.

After discontinuation of study golimumab injections, 101 patients were treated with commercially available biologics through week 268; 36 of these patients experienced an adverse event. Adverse events observed in the 36 patients were similar to events observed during receipt of study medication in the overall trial. Five patients had a serious adverse event reported, including one patient each with histoplasmosis, non-small cell lung cancer, and gastroesophageal reflux disease and two patients with cholecystitis/cholelithiasis.

| **Table S1. Brief summary of patient characteristics and RA medications at baseline of the GO-AFTER trial.** Data presented are mean ± standard deviation [median] or number (%) of randomized patients. | | | |
| --- | --- | --- | --- |
|  | **Placebo→**  **50 mg (±100 mg)** | **Golimumab**  **50 mg (±100 mg)** | **Golimumab**  **100 mg (±50 mg)** |
| Number of randomized patients^1^ | 155 | 153 | 153 |
| Female | 132 (85.2%) | 113 (73.9%) | 122 (79.7%) |
| Age | 54.8 ± 13.07 [54.0] | 53.9 ± 11.47 [55.0] | 53.7 ± 12.26 [55.0] |
| Disease duration (years) | 12.4 ± 9.58 [9.8] | 12.4 ± 9.24 [9.6] | 10.6 ± 7.90 [8.7] |
| CRP (mg/dL) | 2.1 ± 3.16 [1.0] | 2.2 ± 2.97 [0.8] | 2.1 ± 3.38 [0.8] |
| Number of swollen joints (0-66) | 17.5 ± 11.76 [14.0] | 17.8 ± 11.82 [14.0] | 15.4 ± 9.49 [13.0] |
| Number of tender joints (0-68) | 30.0 ± 17.56 [26.0] | 30.6 ± 16.86 [27.0] | 29.1 ± 16.69 [26.0] |
| HAQ-DI score (0-3) | 1.6 ± 0.6 [1.8] | 1.6 ± 0.7 [1.6] | 1.5 ± 0.6 [1.5] |
| DAS28-ESR score (0-10) | 6.2 ± 1.19 [6.3] | 6.3 ± 1.25 [6.3] | 6.1 ± 1.24 [6.1] |
| DAS28-CRP score (0-10) | 5.1 ± 0.99 [5.1] | 5.3 ± 1.05 [5.4] | 5.1 ± 0.92 [5.1] |
| SDAI score (0-100) | 40.9 ± 14.59 [38.4] | 43.2 ± 15.89 [42.1] | 40.7 ± 13.88 [40.3] |
| Previous anti-TNF for RA | 155 (100.0%) | 153 (100.0%) | 153 (100.0%) |
| -Adalimumab | 85 (54.8%) | 72 (47.1%) | 65 (42.5%) |
| -Etanercept | 73 (47.1%) | 76 (49.7%) | 73 (47.7%) |
| -Infliximab | 83 (53.5%) | 64 (41.8%) | 71 (46.4%) |
| Methotrexate use at baseline | 102 (65.8%) | 103 (67.8%) | 100 (65.8%) |
| ^1^ Based on the 445 patients included in efficacy analyses after exclusion of 16 patients at one study site.  *CRP=C-reactive protein, DAS=Disease Activity Score, ESR=erythrocyte sedimentation rate, HAQ-DI=Health Assessment Questionnaire Disability Index, RA=rheumatoid arthritis, SDAI=Simplified Disease Activity Index, TNF=tumor necrosis factor* | | | |

**Figure S1. Patient disposition through week 256.**

**

**Figure S2. Observed clinical efficacy over time through week 256,** including ACR20 **(A),** ACR50 **(B),** DAS28-CRP response **(C),** DAS28-CRP score < 2.6 **(D),** DAS28-CRP score < 3.2 **(E),** SDAI score ≤ 3.3 **(F),** and HAQ-DI improvement **≥ 0.25 (G)**. Observed data are reported among patients receiving methotrexate at baseline who did not discontinue study participation as of week 24. No treatment failure rules were applied, and missing data were not imputed.

*ACR20/50=at least 20%/50% improvement in the American College of Rheumatology responses criteria, CRP=C-reactive protein, DAS28=28-joint Disease Activity Score, HAQ-DI=Health Assessment Questionnaire Disability Index, SDAI=Simplified Disease Activity Index*

**
